# The effect of therapeutic drug monitoring of risperidone and aripiprazole on weight gain in children and adolescents: the SPACe 2: STAR (trial) protocol of an international multicentre randomised controlled trial

**DOI:** 10.1186/s12888-022-04445-6

**Published:** 2022-12-20

**Authors:** Rebecca A. Hermans, Lisa T. Ringeling, Kajie Liang, Sanne M. Kloosterboer, Brenda C. M. de Winter, Manon H. J. Hillegers, Birgit C. P. Koch, Bram Dierckx

**Affiliations:** 1 Department of Child and Adolescent Psychiatry/Psychology, Erasmus University Medical Center, 3000 CB Rotterdam, PO Box 2060, the Netherlands; 2 Department of Hospital Pharmacy, Erasmus University Medical Center, Rotterdam, the Netherlands; 3 Rotterdam Clinical Pharmacometrics Group, Erasmus University Medical Center, Rotterdam, the Netherlands

**Keywords:** Randomised controlled trial, Antipsychotic drugs, Risperidone, Aripiprazole, Children, Adolescents, Autism spectrum disorder, Therapeutic drug monitoring, Pharmacokinetics, Pharmacodynamics

## Abstract

**Background:**

Antipsychotic drugs are an important part of the treatment of irritability and aggression in children with an autism spectrum disorder (ASD). However, significant weight gain and metabolic disturbances are clinically relevant side effects of antipsychotic use in children. In the SPACe study, we showed positive correlations between both risperidone and aripiprazole plasma trough concentrations and weight gain over a 6-month period. The trial SPACe 2: STAR is designed as a follow-up study, in which we aim to research whether therapeutic drug monitoring in clinical practice can prevent severe weight gain, while retaining clinical effectiveness.

**Methods:**

SPACe 2: STAR is an international, multicentre, randomised controlled trial (RCT). One hundred forty children aged 6 to 18 who are about to start risperidone or aripiprazole treatment for ASD related behavioural problems will be randomised into one of two groups: a therapeutic drug monitoring (TDM) group, and a care as usual (CAU) group. Participants will be assessed at baseline and 4, 10, 24, and 52 weeks follow-up. In the TDM group, physicians will receive dosing advice based on plasma levels of risperidone and aripiprazole and its metabolites at 4 and 10 weeks. Plasma levels will be measured in dried blood spots (DBS). The primary outcome will be BMI z-score at 24 weeks after start of antipsychotic treatment. Among the secondary outcomes are effectiveness, metabolic laboratory measurements, levels of prolactin, leptin and ghrelin, extrapyramidal side effects, and quality of life.

**Discussion:**

This will be the first RCT evaluating the effect of TDM of antipsychotic drugs in children and adolescents. Thus, findings from SPACe 2: STAR will be of great value in optimising treatment in this vulnerable population.

**Trial registration:**

ClinicalTrials.gov Identifier: NCT05146245. EudraCT number: 2020–005450-18. Sponsor protocol name: SPACe2STAR. Registered 8 June 2021. Protocol Version 6, Protocol date: 18 august 2022.

**Supplementary Information:**

The online version contains supplementary material available at 10.1186/s12888-022-04445-6.

## Background

An autism spectrum disorder (ASD) is characterised by impairments in social interaction, verbal and non-verbal communication, as well as by different processing of sensory stimuli and stereotypical patterns of behaviour and interests [1]. Apart from these core symptoms, children and adolescents with ASD frequently display behaviour such as temper tantrums, aggression and self-harm [2]. Indeed, a Dutch study showed that almost half of the children and adolescents diagnosed with ASD suffered from a comorbid behavioural disorder [3]. Pharmacological interventions are an important part of the multimodal treatment of these comorbid behavioural problems. National and international guidelines recommend antipsychotic drugs as agents of first choice [4, 5]. Our research has shown that antipsychotic drug use in the Netherlands is high, with 8.5 out of every 1000 children and adolescents using antipsychotics [6]. Among these drugs, risperidone and aripiprazole are most often prescribed.

Previously, few studies have adequately monitored long term antipsychotic safety profiles, though evidence indicates that extrapyramidal symptoms, cardiovascular and especially metabolic abnormalities are clinically relevant adverse effects in younger patients, with the individual agents differing in their propensity to induce different side effects [7, 8]. In atypical antipsychotics, the most prescribed class of antipsychotics in children and adolescents, metabolic abnormalities are of particular concern [8]. In short term studies in children, weight gain amounted to an approximately 10% increase when compared to baseline [9]. In addition, antipsychotics have been reported to increase triglycerides by up to 45% [10]. Different studies showed an increased risk of type 2 diabetes in children and adolescents receiving antipsychotic medication [11, 12]. Moreover, childhood obesity is highly predictive of adulthood obesity [13], and when childhood obesity persists in adulthood, these adults are at an even higher risk of type 2 diabetes, hypertension, dyslipidaemia, and atherosclerosis compared to persons with adult-onset obesity [14].

In adults, there is evidence that therapeutic drug monitoring (TDM), i.e. the quantification of serum drug concentrations for dose optimisation, can help in maximising clinical efficacy while minimising the risk of side effects [15]. Several studies have shown a correlation between risperidone plasma levels and both clinical efficacy and extrapyramidal side effects [16], but studies targeting the correlation between drug plasma levels and indices of metabolic side effects in adults are lacking. Previously, in children and adolescents, only a few studies have been performed investigating the relationship between antipsychotics and response or side-effects. However, all of these studies had suboptimal study designs, as drug sampling was not performed under standardised protocols or due to a retrospective design.

For this reason, we carried out the SPACe study (Netherlands Trial Register: 6050), in which we researched the relationship between antipsychotic drug plasma levels and metabolic side effects in children. Forty-two children who used risperidone and 23 children who used aripiprazole were included, for whom we developed population pharmacokinetic models. These population pharmacokinetic models showed that higher trough levels of aripiprazole and risperidone predicted higher BMI z-scores over a 6-month period. Following this finding, therapeutic windows have been defined, where sufficient treatment effect and acceptable weight gain is expected to be achieved [17] (Hermans RA, Sassen SDT, Kloosterboer SM, Reichart CG, Kouijzer MEJ, de Kroon MMJ, et al. Precision dosing of aripiprazole in children and adolescents: linking blood levels to weight gain and effectiveness. Submitted. 2022).

While we now know that there is a strong correlation between antipsychotic plasma levels and metabolic side effects in children, we still need to investigate whether application of TDM in clinical practice is indeed able to reduce the severity or possibly even the occurrence of these side effects, while retaining clinical effectiveness. With this objective, we designed the randomised controlled trial SPACe 2: STAR. We hypothesise that children receiving TDM will have a lower side effect burden, as evidenced by less weight gain, when compared to children receiving care as usual, without compromising treatment effectiveness.

## Methods and design

**Fig. 1:**
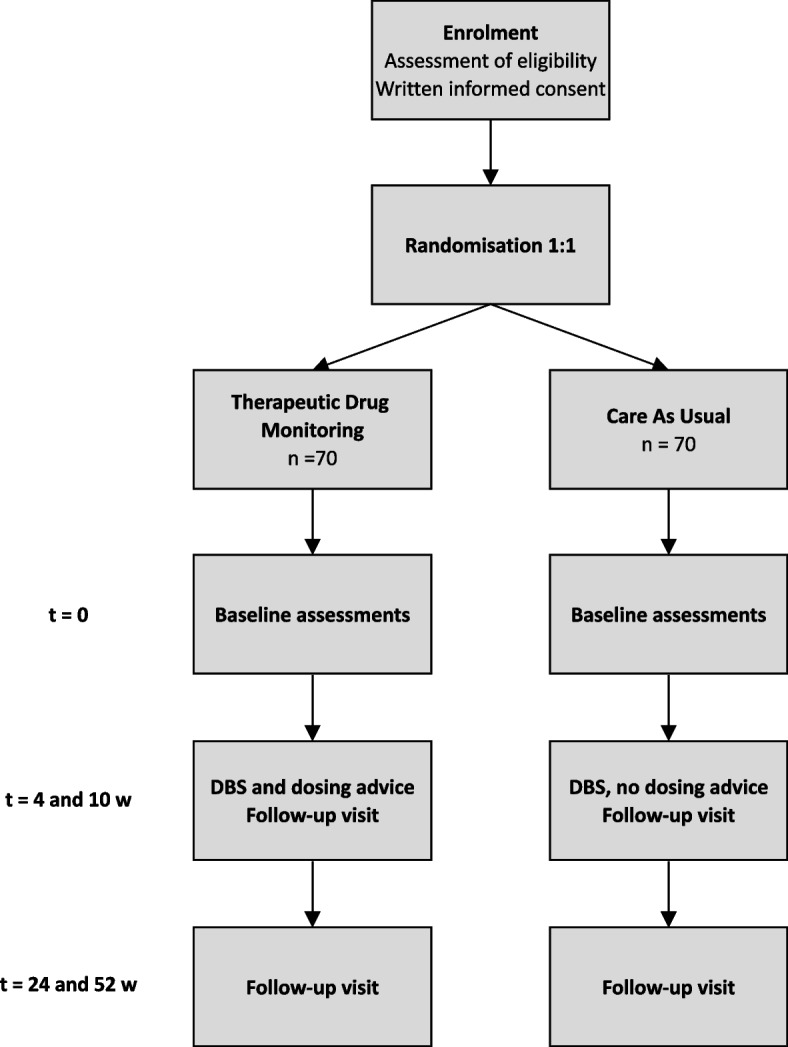
Diagram of SPACe 2: STAR trial design

SPACe 2: STAR is an international, multicentre, randomised controlled trial (RCT) in which we aim to research whether TDM of risperidone and aripiprazole is able to prevent or mitigate weight gain and other side effects, while retaining clinical effectiveness in children and adolescents with ASD and comorbid behavioural problems. An overview of the trial design is provided in Fig. 1 and Fig. S1.

### Participants

Both in- and outpatients of the participating centres are eligible for inclusion if they are 6 to 18 years old, with a documented clinical diagnosis of autism spectrum disorder according to DSM IV or DSM V and comorbid behavioural problems, and will start treatment with risperidone or aripiprazole. An inclusion scheme is followed (Table S1), in which per hospital the anticipated inclusion rate is presented.

Exclusion criteria are: (1) diabetes type I or II, (2) congenital or acquired syndrome associated with changes in appetite, body weight or lipid profile (e.g. Prader Willi), (3) known Long QT syndrome, and (4) pregnancy.

#### Withdrawal

Participants can withdraw from the study at any time, without having to state a reason and without any consequences. The investigator can decide to withdraw a subject from the study for urgent medical reasons, if a subject is pregnant, or if TDM is applied for a participant in the CAU group.

When a patient discontinues antipsychotic treatment before the end of the study, the reason for discontinuation is extracted from the patient’s medical file by the researcher. Depending on the phase of follow-up, close-out measurements will be taken as well.

### Informed consent

According to Dutch law, both parents or a legal representative need to provide informed consent for patients aged 6–11. The child will be informed according to their level of comprehension, using an adapted/customized patient information folder with graphics. For patients aged 12–16, both the consent of the child and both parents or legal representative is needed. For patients aged 16 or older, only the patient needs to sign for informed consent.

The treating physicians have extensive experience in working with vulnerable children, and will additionally be instructed before start of the study about recognition of expressions of resistance. When they doubt whether a child shows resistance, this will be discussed with the parents and if needed with the researcher. If at any time the minor offers resistance to a specific study measurement (other than standard care), permission for further participation in these measurements is withdrawn.

### Allocation

Participants will be randomly assigned in a 1:1 ratio to one of two groups: the therapeutic drug monitoring group or care as usual group. We will use the randomisation module from Castor EDC to apply block randomisation with stratification for type of antipsychotic drug (risperidone or aripiprazole), for use of ADHD medication (methylphenidate, dexamphetamine or atomoxetine), and per institute. Randomisation will be unblinded.

### Pharmacokinetic sampling

Blood sampling for antipsychotic quantification will be performed using the dried blood spot (DBS) method, where blood is obtained by means of a fingerprick, and dried on a filter paper. We successfully validated this method for the simultaneous quantification of risperidone and its metabolite 9-OH-risperidone as well as for aripiprazole and its metabolite dehydro-aripiprazole [18, 19]. We previously applied DBS in the SPACe study, and found that this minimally invasive procedure is well tolerated by children with ASD [20]. DBS samples will be obtained for dosing advice at 4 and 10 weeks after start of antipsychotic treatment. All samples will be analysed within 1 week in the laboratory of the Department of Hospital Pharmacy in Erasmus University Medical Center. Antipsychotic drug concentrations measured in DBS will then be converted to estimated plasma concentrations (EPCs), using the formulas devised in our clinical validation study [18].

### Intervention: dosing advice

In the TDM intervention group, risperidone or aripiprazole is started according to local and national guidelines and at the discretion of the attending physician. At 4 and 10 weeks, physicians will be given a tailored advice for dose adjustment, guided by online software platform InsightRX® (version 1.39.7, San Francisco, California). InsightRX incorporates the principles of quantitative pharmacology and machine learning to provide an individualised understanding of a patient’s response to treatment. InsightRX will initially be fed with the pharmacokinetic (PK) models for risperidone and aripiprazole, which we developed in the SPACe study. Based on estimated plasma concentrations (EPCs) and patient-specific parameters (weight and date of birth for users of risperidone or aripiprazole, and also height and albumin level for users of aripiprazole), InsightRX will be able to extrapolate expected sum (parent drug + metabolite) trough levels of risperidone and aripiprazole at different dosages. The extrapolated expected sum trough levels within the target values of risperidone and aripiprazole will lead to theoretical optimal individual doses. Risperidone and aripiprazole target values are provided in Table 1. Adherence to or deviation from dosing advice will be registered in the electronic Case Report File (eCRF).

**Table 1:** Target values of trough levels of risperidone and aripiprazole

Antipsychotic drug	Laboratory target	Target values
**Risperidone**	Sum (risperidone + 9-hydroxyrisperidone) trough level	Therapeutic window: 3.5–7 μg/L Optimal target: 5.25 μg/L
**Aripiprazole**	Sum (aripiprazole + dehydro-aripiprazole) trough level	Therapeutic window: 13.6–60 μg/L

In the CAU group, risperidone or aripiprazole is started and titrated according to local and national guidelines and at the discretion of the attending physician. Plasma levels will be measured to allow for post hoc comparison between the groups, but no dosing advice is given and physicians cannot see the measured plasma levels.

### Study parameters and assessments

The text below describes study outcomes and related assessments. Table 2 provides an overview of all assessments and time points.

**Table 2:** Data collection across the trial

Assessment	Week				
	0	4	10	24	52
Primary outcome					
Weight, height	x	x	x	x	x
Secondary outcomes					
ABC	x	x	x	x	x
PedsQL + EQ-5D-Y	x	x	x	x	x
EQ-5D-Y	x	x	x	x	x
ESS	x	x	x	x	x
Laboratory measurements	x			x	x
Blood pressure	x	x	x	x	x
AIMS + MSAS	x	x	x	x	x
CGI	x	x	x	x	x
Other					
DBS + medication diary		x	x		
Treatment adherence		x	x	x	x
Treatment questionnaire	x	x	x	x	x
Medication overview pharmacy					x
Eating/hunger questionnaire	x	x	x	x	x
DNA sampling	x				
Demographic variables	x				

#### Primary outcome

Difference between the groups in change in BMI z-scores after 6 months of treatment. BMI z-scores are measures of relative weight adjusted for child age and sex, to track relative weight status through the treatment. The change in BMI z-scores is a first indicator of developing metabolic problems. We chose a 6 month follow up time for the primary outcome, because in the SPACe study we saw rapid weight gain in the first 15 weeks of treatment, followed by plateauing [21].

#### Secondary outcomes

##### Weight gain

Difference between the groups in change in BMI z-scores after 12 months of treatment.

##### Effectivity

Difference in scores on the irritability subscale of the Aberrant Behaviour Checklist (ABC-I), a parental symptom checklist for assessing problem behaviour in children. This is the gold standard for measuring the effect of treatment on aggression and irritability in children with autism spectrum disorder [22]. Effectivity is defined as a reduction in ABC-I of 25%.

Difference in Clinical Global Impression Scale (CGI), as filled in by treating physician is also measured [23].

##### Quality of life

Quality of life will be measured through two questionnaires, which will be filled in by parents or caretakers and, depending on age and capability, by children as well:

- Paediatric Quality of Life Inventory (PedsQL): a modular instrument aimed at measuring health-related quality of life in children and adolescents. It showed excellent reliability and validity in individuals with autism spectrum disorder [24].
- EuroQol-5D Youth (EQ-5D-Y): a descriptive system for health-related quality of life states in five dimensions, plus a visual analogue scale [25].

##### Secondary safety parameters

We will compare the standard laboratory parameters for monitoring antipsychotic metabolic side effects, namely levels of glucose, cholesterol, lipoproteins and triglycerides. Additionally, further endocrine parameters, including prolactin, ghrelin and leptin, will be measured.

Extrapyramidal symptoms (EPS) will be assessed by treating physicians with the Abnormal Involuntary Movement Scale (AIMS), which measures tardive dyskinesia, complemented with the items for elbow rigidity, wrist rigidity or fixation of position, and tremor from the Modified Simpson-Angus Scale (MSAS), and assessment of cogwheel rigidity [26, 27].

Sedation caused by antipsychotic treatment will be assessed through the Epworth Sleepiness Scale (ESS), to be filled in by parents or child [28]. Lastly, blood pressure will be measured at all study visits.

#### Other study parameters and assessments

DNA will be sampled to determine CYP metaboliser status as well as analyse possibly relevant single nucleotide polymorphisms, which will be investigated as potential moderators between antipsychotic trough plasma levels and metabolic side effects.

Physicians will be asked to estimate treatment adherence on a visual analogue scale of 0 to 100. Personal characteristics and data on health, non-pharmacological interventions and concomitant medication use will be extracted from the participant’s file and gathered through a short questionnaire. Participants will also be asked for permission to obtain a medication overview from their community pharmacy. Use of risperidone or aripiprazole on the day before and day of DBS sampling will be entered in a medication diary.

Lastly, in order to learn more about the mechanisms of weight gain associated with antipsychotic use, we will administer a short questionnaire on hunger, satiety and eating behaviour, which was devised for this study in collaboration with the department of Endocrinology at Erasmus University Medical Center.

### Statistical analyses

#### Primary outcome

BMI scores for children will be standardised for age and sex using Dutch reference values to obtain BMI z-scores (continuous). The change in BMI z-scores in children receiving TDM will be compared to children receiving CAU during the first 6 months of treatment by means of repeated Measure ANOVA. Age, sex, weight at start of treatment, treatment with antipsychotic medication within the last 6 months, class of comedication, CYP status will all be entered as covariates.

The analysis will be performed according to the intention-to-treat (ITT) principle. The ITT population will consist of all patients who have been randomised, irrespective of withdrawals, dropouts or other reasons for failing to complete the study. In addition, per protocol analyses will be done. For the per protocol analyses, only randomised patients who at least had one TDM advice for dose adjustment of study medication will be entered into the analysis. When participants are lost to follow-up after a plasma concentration has been obtained, data will be analysed according to the last observation carried forward principle. For covariates, missing values will be addressed with multiple imputation.

#### Secondary outcomes

The same approach as described above will be taken for the secondary outcomes (all continuous variables), comprising of BMI z-score at 1 year, the irritability subscale of the ABC, EQ-5D-Y, PedsQL, CGI, AIMS, systolic and diastolic blood pressure, levels of glucose, cholesterol, lipoproteins, triglycerides, prolactin, ghrelin and leptin.

### Sample size calculation

A 5% change in body weight is generally seen as clinically significant [29]. For children, 5% gain of bodyweight on top of their normal growth trajectory amounts to an increase in BMI z-score of about 0.5 over a 6-month period. Using a conservative power analysis for basic between groups’ differences, with a power of 80% and an alpha of 0.05, including 2 × 64 children allows us to detect medium clinical effects. Moreover, use of the repeated measures approach is likely to allow us to detect smaller effect sizes as well. In the SPACe study, we found an overall event rate of 40% significant increase in BMI z-score. 2 × 64 patients affords us enough power to detect a halving of the event rate in the active group. Allowing for 10% dropout, we aim to include a total of 140 patients.

### Data management and monitoring

Questionnaires will be disseminated through a secured online server of the hospital and health data collected by researchers will be stored in an eCRF. Patient data will be pseudo-anonymised through allocation of subject identification codes (SICs). Data will be stored on a secure drive with access only granted to (principal) investigators, monitors or the Health and Youth Care Inspectorate. Paper data will be stored in locked filing cabinets. Data handling will be in line with the General Data Protection Regulation (GDPR).

Because this trial has a high risk of low complications, an independent monitor will visit each study site every 6 months. 25% of all cases are randomly selected for verification by the independent monitor. Informed consent, source data and reported serious adverse events (SAEs) are reviewed for errors.

### Serious adverse events

Serious adverse events (SAEs) will be reported to the local medical ethics committee through an online platform within 7 days of first knowledge for SAEs that result in death or are life threatening. All other SAEs will be reported within a period of maximum 15 days after the sponsor has first knowledge of the serious adverse events.

## Discussion

Risperidone and aripiprazole are frequently prescribed to children and adolescents, despite regular occurrence of side effects. SPACe 2: STAR will be the first randomised controlled trial of TDM of antipsychotic drugs in this population. The previously performed SPACe study, our research consortium and the simulation study we carried out yielded results that support our hypothesis of the added benefit of TDM and provide a strong basis for this trial. We will perform TDM in the first months of antipsychotic treatment, the critical time when most weight gain occurs, in hopes of preventing metabolic side effects in both the short and long term.

One of the biggest challenges in carrying out this study will be the recruitment of participants. We hope to have tackled this by including patients using the two most frequently prescribed antipsychotic drugs for children and adolescents and by including a high number of participating centres.

A possible limitation of our study lies in the nature of giving a non-binding dosing advice. There is a risk that physicians will decide to deviate from our laboratory-based advice because of clinical findings, e.g. a lack of effectiveness at a low dose. We have chosen this style of intervention to accurately represent TDM in clinical practice where deviation from therapeutic windows is possible, and we will document adherence to dosing advice. Should frequent deviation from dosing advice occur, this would be an important observation on the clinical usability of TDM for this indication. Furthermore, we realise that a double-blind study design may lead to a higher quality of evidence, as the possibility of bias is minimized. To enable double-blind research in our study, physicians would need to be blinded when giving dose recommendations. Since only the TDM group receives dosing advice, we are of the opinion that a double-blind study design is not feasible.

In conclusion, SPACe 2: STAR is a unique, innovative clinical trial, which will aim for a level of evidence that is rarely achieved. The outcomes will provide valuable information on optimising antipsychotic drug treatment in the paediatric population.

## Supplementary Information

**Additional file 1: Figure S1.** SPIRIT Figure of the timeline of SPACe 2: STAR.

**Additional file 2: Table S1.** Participating medical centres and the inclusion estimations.

## Data Availability

The datasets generated during and/or analysed during the current trial are available from the corresponding author on reasonable request after publication. The data will need to be requested in the context of research approved by a medical ethical committee and will need to follow the General Data Protection Regulation.

## Abbreviations

ABC-I: Irritability subscale of the Aberrant Behaviour Checklist
AIMS: Abnormal Involuntary Movement Scale
ASD: Autism spectrum disorder
BMI: Body mass index
CAU: Care as usual
CGI: Clinical Global Impression scale
DBS: Dried blood spot
DSM: Diagnostic and Statistical Manual of Mental Disorders
eCRF: Electronic case report file
EPC: Estimated plasma concentration
EPS: Extrapyramidal symptoms
EQ-5D-Y: EuroQol-5D Youth
ESS: Epworth Sleepiness Scale
GDPR: General Data Protection Regulation
ITT: Intention to treat
MSAS: Modified Simpson-Angus Scale
PedsQL: Paediatric Quality of Life Inventory
PK: Pharmacokinetic
RCT: Randomised controlled trial
SAE: Serious adverse event
SIC: Subject identification code
TDM: Therapeutic drug monitoring
