# Supplementary material for: The effect of therapeutic drug monitoring of risperidone and aripiprazole on weight gain in children and adolescents: the SPACe 2: STAR (trial) protocol of an international multicentre randomised controlled trial
Source: BMC Psychiatry. 2022 Dec 20;22:814. doi: 10.1186/s12888-022-04445-6 (PMC9769061; doi:10.1186/s12888-022-04445-6)
Supplement: Supplementary file 1 — Additional file 1: Figure S1. SPIRIT Figure of the timeline of SPACe 2: STAR. [file 12888_2022_4445_MOESM1_ESM.docx]

|  | **STUDY PERIOD** | | | | | |
| --- | --- | --- | --- | --- | --- | --- |
|  | **Enrolment** | **Allocation** | **Post-allocation** | | | **Close-out** |
| **TIMEPOINT** | **t=-1w-0** | **t=0** | **t=4w** | **t=10w** | **t=24w** | **t=52w** |
| **ENROLMENT** | | | | | | |
| **Eligibility screen** | X |  |  |  |  |  |
| **Informed consent** | X |  |  |  |  |  |
| **Allocation** |  | X |  |  |  |  |
| **INTERVENTIONS** | | | | | | |
| **DBS/Dosing advice** |  |  | X | X |  |  |
| **ASSESSMENTS** | | | | | | |
| **Demographics** | X | X |  |  |  |  |
| **Physician observations** |  | X | X | X | X | X |
| **Questionnaires** |  | X | X | X | X | X |
| **Laboratory measurements** |  | X |  |  | X | X |
