# Supplementary material for: The effect of therapeutic drug monitoring of risperidone and aripiprazole on weight gain in children and adolescents: the SPACe 2: STAR (trial) protocol of an international multicentre randomised controlled trial
Source: BMC Psychiatry. 2022 Dec 20;22:814. doi: 10.1186/s12888-022-04445-6 (PMC9769061; doi:10.1186/s12888-022-04445-6)
Supplement: Supplementary file 2 — Additional file 2: Table S1. Participating medical centres and the inclusion estimations. [file 12888_2022_4445_MOESM2_ESM.docx]

**Appendix 2**

**Table S1.** Participating medical centres and the inclusion estimations.

| **Study sites** | **Expected enrolment** |
| --- | --- |
| **Erasmus Medical Center (Rotterdam)** | 30 |
| **Yulius GGZ (Barendracht)** | 15 |
| **GGZ Breburg Groep (Breda)** | 15 |
| **GGZ Delfland (Delft)** | 15 |
| **Leiden University Medical Center** | 15 |
| **UZ Leuven** | 10 |
| **University Clinic Wurzburg** | 10 |
| **University Clinic Tubingen** | 10 |
| **Specialist Hospital Mariaberg** | 10 |
| **‘s Heeren Loo (Hoofddorp)** | 10 |
| **Accare (Groningen)** | 10 |
